# EZH2 as a therapeutic target for multiple myeloma and other haematological malignancies

**DOI:** 10.1186/s40364-018-0148-5

**Published:** 2018-12-07

**Authors:** Rosemarie Tremblay-LeMay, Nasrin Rastgoo, Maryam Pourabdollah, Hong Chang

**Affiliations:** 1 Laboratory medicine program, Toronto General Hospital, University Health Network, University of Toronto, 200 Elizabeth Street, 11th floor, Toronto, ON M5G 2C4 Canada; 2 Division of Molecular and Cellular Biology, Toronto General Research Institute, Toronto, Canada; 3 Department of Talent Highland, First Affiliated Hospital of Xi’an Jiao Tong University, Xian, China

**Keywords:** EZH2, Multiple myeloma, Targeted therapy, Drug resistance

## Abstract

Enhancer of zeste homolog 2 (EZH2) is a histone methyltransferase that is of great interest in human cancer. It has been shown to have a dual nature, as it can act as a gene repressor or activator. Studies have highlighted the various roles of EZH2 in the pathophysiology of multiple myeloma (MM). It was also shown to have a role in the development of drug resistance in MM. There are several ongoing clinical trials of EZH2 inhibitors in haematological malignancies. Pre-clinical studies have provided a rationale for the therapeutic relevance of EZH2 inhibitors in MM. This paper reviews the evidence supporting the role of EZH2 in MM pathophysiology and drug resistance, with an emphasis on interactions between EZH2 and microRNAs, as well as the prognostic significance of EZH2 expression in MM. Furthermore, results from the pre-clinical studies of EZH2 inhibition in MM and currently available interim results from clinical trials of EZH2 inhibitors in haematological malignancies are presented. Preliminary data exploring anticipated mechanisms of resistance to EZH2 inhibitors are also reviewed. There is therefore strong evidence to support the relevance of targeting EZH2 for the treatment of MM.

## Background

Enhancer of zeste homolog 2 (EZH2) is a catalytic subunit of the initiation complex polycomb repressive complex 2, which is part of the polycomb group proteins. It is a histone methyltransferase that tri-methylates histone H3 at Lys 27 (H3K27me3) and silences target genes involved in various functions such as cell cycle, cell proliferation, cell differentiation. When it is dysregulated in tumorigenesis, it can affect cell proliferation and apoptosis, epithelial to mesenchymal transition and invasion, and drug resistance [1]. EZH2 expression in tumoral cells can modulate immune response and immunotherapy. Expression in immune cells from the tumor microenvironment can affect tumor progression and that it has direct roles on T cell response. A recent review highlighted the evidence supporting the role of EZH2 in tumor immunity in cancer [1]. This might eventually prove to be of relevance in the context of MM, as tumor microenvironment plays an important role in MM pathogenesis. EZH2 has also been identified as critical to cancer stem cell expansion and maintenance in various malignancies through its action on BMP signalling, AKT/EZH2/STAT3 signaling, Wnt/β-catenin signalling and Notch signalling. It also interacts with genes related to stemness, such as p16, p19, E-cadherin or c-Myc, as well as microRNAs (miRNAs) involved in regulating cancer stem cells [2]. It has a dual nature, as it can act as a gene repressor or activator. EZH2 can interact with transcription factors and co-factors to activate gene expression. It is a co-activator of STAT3 and androgen receptor; it interacts with β-catenin and oestrogen receptor α, linking Wnt and oestrogen pathways; it interacts with RelA/RelB to activate NF-κB; it interacts with PCNA-associated factor, thereby promoting activation of Wnt target genes. It can also silence gene expression by methylating both histone and non-histone proteins (GATA4, RORα) [3]. The reader is referred to the recent paper by Gan and colleagues for a more detailed review of the transcriptional, post-transcriptional and post-translational regulation of EZH2 [1].

EZH2 inhibition has been identified as a promising strategy for the treatment of haematological malignancies. Follicular lymphoma (FL) and germinal-centre B-cell like (GCB) diffuse large B-cell lymphoma (DLBCL) in particular were found to frequently harbour *EZH2* gain-of-function mutations that renders them particularly sensitive to EZH2 inhibition. The most frequently mutated site is Y641 (also known as Y646) in the SET domain of EZH2. Mutations of Y641 are found in FL, GBC DLBCL, high grade B-cell lymphoma with concurrent BCL2 and MYC rearrangement, but not activated B-cell (ABC) DLBCL [4–6]. The mutated allele is associated with a wild-type allele and evidence suggests the pathogenesis may be dependent on the interaction between mutated and non-mutated alleles [7]. Other hotspot mutations include A677G [8], A682G and A692V [9]. Mutations at Y646, A682 or A692 were found in 27% FL in a series of 366 patients and were maintained in the majority of patients with transformation [10]. Myeloid neoplasms, on the other end, tend to have missense, frameshift or non-sense mutations leading to loss-of-function [11].

In contrast, while EZH2 overexpression is reported in MM [12–14], recurrent mutations of EZH2 are not significantly observed [15, 16]. Epigenetic mechanisms appear to be involved in the overexpression of EZH2 in MM [17], rather than the gain-of-function mutation observed in follicular lymphoma. Evidence suggests that EZH2 inhibition is a potential therapeutic strategy to treat MM. Current knowledge regarding the potential mechanisms of pathogenesis of MM involving EZH2 will be reviewed, as well as the prognostic significance of EZH2 expression, pre-clinical studies of EZH2 inhibition in MM, clinical trials ongoing in haematological malignancies and anticipated mechanisms of resistance to EZH2 inhibition.

### Role of EZH2 in MM

**Fig. 1:**
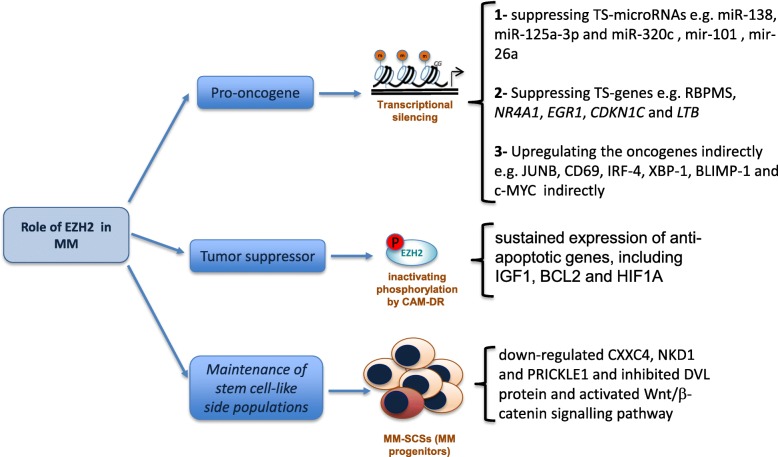
Roles of EZH2 in multiple myeloma. EZH2 can act as a pro-oncogene, through transcriptional silencing of tumor suppressors (TS) or indirect upregulation of oncogenes. EZH2 can also act as a tumor suppressor by inactivating the phosphorylation by cell adhesion-mediated drug resistance (CAM-DR) mechanisms, leading to sustained expression of anti-apoptotic genes. EZH2 is also involved in the maintenance of multiple myeloma stem cell-like side populations (MM-SCSs)

EZH2 has been shown to have both tumor suppressor and pro-oncogene properties depending on the cancer type [3]. While most of the reports had suggested that EZH2 might act as an oncogene, a few studies have demonstrated the tumor suppressor role of EZH2 in the context of MM. Studies also support a role for EZH2 in mechanisms of drug resistance and the maintenance of stem cell-like side populations in MM (see Fig. 1).

#### Pro-oncogene

Early reports highlighted the oncogenic activity of EZH2 in MM. Croonquist and colleagues showed that EZH2 expression increases with proliferation of MM cell lines and that blocking EZH2 expression with short interference RNA inhibits MM cell growth [13]. They also showed that ectopic EZH2 expression could render IL-6-dependent cell lines growth factor independent. EZH2 overexpression was sufficient to induce tumor formation in mice, suggesting it is a true oncogene [13]. Data from pre-clinical studies of EZH2 inhibitor E7438 in MM cell lines and mice model showed that EZH2 inhibition causes a global reduction in H3K27me3 methylation in a dose dependent manner, which in turn led to re-expression of tumor suppressor genes in some of the cell lines. Many of these upregulated genes were related to adhesion and a more epithelial phenotype, which correlated with a more spindle-like and adherent phenotype of MM cells [18]. GSK126, an EZH2 inhibitor, was found to induce apoptosis in MM cells in a mitochondrial pathway-dependent manner, characterized by cleavage of MCL-1 by active caspase-3 [19]. Similarly, EPZ-6438-induced apoptosis could be partially rescued by a pan-capsase inhibitor [20]. EPZ005687 and UNC1999 induced apoptosis by upregulating cyclin-dependent kinase inhibitors associated with the removal of the inhibitory H3K27me3 mark at their gene loci [21]. RNA sequencing showed that UNC1999 targets genes including *NR4A1*, *EGR1*, *CDKN1C* and *LTB* (a component of NF-κB). The molecule induced *NR4A1* upregulation, which in turn suppressed *MYC*, resulting in growth suppression. This effect was enhanced by the combination with bortezomib [22]. It was also shown to up-regulate transcripts related to apoptosis, Wnt, ID, MAPK, insulin signalling pathways and cellular differentiation [23], while it reduces the levels of MM-associated oncogenes JUNB, CD69, IRF-4, XBP-1, BLIMP-1 and c-MYC, presumably by modulating the expression of miRNAs [23, 24]. EPZ-6438 was found to target known polycomb target genes, genes enriched in H3K27me3 histone mark, genes associated with DNA methylation in MM, TP53 and RB1 target genes [20].

Interestingly, knockdown of *EZH1* was found to induce growth inhibition of MM cells, although the effect was less significant than with *EZH2* knockdown, suggesting that both EZH2 and EZH1 play a role in MM pathogenesis. Double knockdown of *EZH1* and *EZH2* induced a more significant cell death than knockdown of either gene alone, suggesting that dual EZH1/2 inhibitors might be of interest in MM [22].

#### Drug resistance

EZH2 has been implicated in mechanisms of cell adhesion-mediated drug resistance, a term that refers to the fact that MM cells are less sensitive to chemotherapeutic agents due to interactions with the bone marrow stromal cells. Kikuchi and colleagues found that direct contact with bone marrow stromal cells caused phosphorylation-mediated EZH2 inactivation, leading to H3K27 hypomethylation and expression of antiapoptotic genes. Functional assays revealed that adhesion signals activated the PI3K/Akt pathway to induce the phosphorylation of EZH2, resulting in the activation of antiapoptotic genes such as *IGF1*. A feedback loop occurs since secreted IGF-1 activates the IGF-1/IGF-1R pathway, which in turn further enhances the activation of the PI3K/Akt pathway. Small molecule inhibitor of IGF-1R could promote EZH2 dephosphorylation in vitro and in vivo. Bortezomib induced H3K27 hypermethylation by perturbing EZH2 phosphorylation even in the presence of stromal cells, thereby reversing cell adhesion-mediated resistance [25]. A study showed that proteasome inhibitor bortezomib reduces EZH2 protein and mRNA expression in dose and time-dependent manner, however in resistant cell lines EZH2 protein was not downregulated, suggesting a possible role of EZH2 in bortezomib cytotoxicity. They determined that this effect is mediated by the accumulation of cyclin-dependent kinase inhibitors p21 and p27, leading to reduced RB phosphorylation and inactivation of the E2F family, thereby downregulating *EZH2* [22].

Immunomodulators (IMiD) are also part of the standard of care for MM, but resistance can occur. Dimopoulos and colleagues showed that acquired IMiD resistance is associated with an increase in genome-wide DNA methylation and downregulation of SMAD3. Treatment of resistant MM cell lines with a combination of 5-azacytidine (5-Aza) and EZH2 inhibitor EPZ-6438 could restore SMAD3 expression, which led to re-sensitization to IMiD [26].

#### Maintenance of stem cell-like side populations

Side populations have tumorigenic potential and share features with stem cells such as the capacity for self-renewal. They are resistant to chemotherapeutic drugs; therefore they play a major role in relapse [27]. Nara and colleagues isolated side populations from the main populations in primary patient samples and MM cell lines [28]. They identified differentially expressed genes that were cell cycle and mitosis-related, proteasome-related or polycomb-related. EZH2 was part of these genes, and its expression was significantly higher in side populations in 6/8 patient samples and 4/5 MM cell lines. Bortezomib could markedly reduce the percentage of side population cells in MM cell lines and also decreased side population cell colonies, which was associated with a downregulation of both Aurora B and EZH2 [28]. Another study showed that inhibition of EZH2 or transfection with a methyltransferase activity-dead EZH2 mutant could significantly reduce the percentage of side population cells in MM cell lines. The authors also showed that loss of EZH2 methyltransferase activity down-regulated the Wnt/β-catenin signalling pathway, which is known for its role in the stemness of cancer stem cells, via upregulation of CXXC4, NKD1 and PRICKLE1 with subsequent inhibition of DVL protein [19]. These results indicate that EZH2 plays a role in the maintenance of stem cell-like side populations in MM.

### Interactions between EZH2 and microRNAs in MM

Yan and colleagues previously published a comprehensive review of the epigenetic regulation of EZH2 [3]. Several miRNAs have been shown to post-transcriptionally modulate the expression of EZH2. The majority act as suppressors of *EZH2*, however miR-21 and miR-210 have been shown to act as pro-oncogenes when co-expressed with *EZH2* [3].

MiR-29b was identified as a relevant tumor suppressor in MM. Inhibition of EZH2 using DZNep, GSK343 or EPZ005687 decreased H3K27me3 levels and triggered miR-29 upregulation that was functionally active. EZH2 specifically binds to miR-29a/b-1 promoter regions. Co-inhibition of EZH2 and miR-29b abrogated the anti-MM activity of EZH2 inhibitors, suggesting that miR-29b is essential for their activity [29].

EZH2 is also known to interact with tumor suppressor miRNAs, which contributes to MM cell proliferation and drug resistance [30]. EZH2 has also been shown to interact with miR-101 in MM [31]. Alzrigat and colleagues analyzed mature miRNA expression in a MM cell line following treatment with EZH2 inhibitor UNC1999 and found a dowregulation of oncomiRNAs described in MM, such as miR-17-92 cluster, miR106b-25 cluster and Let-7 family. There was also upregulation of miRNAs with potential tumor suppressor functions in MM, such as miR-125a, miR-198, miR-223-3p, miR-320c, miR-601, miR-630, miR-765, miR-877-5p and miR-1290. Using chromatin immunoprecipitation assays, they confirmed that miR-125a and miR-320c, which are predicted to regulate MM oncogenes IRF-4, XBP-1 and BLIMP-1, are targets of EZH2 in MM [24].

Our team recently demonstrated that EZH2/miR-138 axis is involved in MM drug resistance. The luciferase assay confirmed EZH2 as a direct target of miR-138. It was also shown that overexpression of miR-138 could re-sensitize resistant cells to bortezomib and was associated with reduced proliferation and increased apoptosis. Furthermore, RNA-binding protein with multiple splicing (RBPMS) gene was found as a downstream target of EZH2 that which is downregulated in resistant cell lines and upregulated in EZH2 inhibited/silenced or miR-138 overexpressing cells. Functional study of RBPMS silenced cells validated the tumor suppressor activity of RBPMS in MM. The overall findings indicate that EZH2 regulates tumor cell growth by repressing RBPMS. The therapeutic potential of miR-138 in MM was explored in an animal model of drug-resistant MM cells and found out that combination treatment of miR-138 mimics and BTZ could significantly prolong the overall survival without any significant weight loss, whereas either treatment alone only resulted in a modest improvement in survival compared to the control. These experiments confirmed that targeting EZH2 with miR-138 sensitizes resistant cells to bortezomib [17].

### Prognostic significance EZH2 expression in MM and other haematological malignancies

In a systematic review with meta-analysis, pooled results for a variety of solid malignancies showed that high EZH2 expression was found to be significantly associated with poor overall survival and disease free survival [32]. Similar results were observed in MM. Pawlyn and colleagues analyzed public datasets from clinical trials with different treatment regimen and found that high EZH2 mRNA expression was higher in symptomatic MM patients versus smoldering MM patients [21]. It was also an independent prognostic factor for shortened progression-free survival and overall survival, and correlates with high-risk gene expression profile [21]. Another study of independent cohorts of untreated MM patients also confirmed that *EZH2* expression is associated with outcome. They also found significant overexpression of EZH2 in patients with del(17p) and 1q21 gain [20].

In a recent study by our group, analysis of public datasets GSE6477 and GSE26760 showed that EZH2 expression was significantly upregulated in relapsed patients compared to newly diagnosed patients and was correlated to the progression of the disease. Analysis of datasets GSE31161 and GSE82307 with paired samples at baseline/newly diagnosed versus relapse also confirmed overexpression in relapsed MM [17]. EZH2 protein expression by immunohistochemistry (IHC) was also evaluated in a cohort of 67 newly diagnosed MM patients. High expression was associated with significantly shorter median progression free survival and overall survival compared to patients with low expression [17]. Multivariate analysis accounting for β2-microglobulin and haemoglobin levels confirmed EZH2 was an independent predictor of worse overall survival (*p*-value 0.0192, HR = 3.421, 95% CI (1.222–9.572)) (Additional unpublished data).

### EZH2 inhibition in MM

#### Antitumoral effects

A number of pre-clinical studies have demonstrated the relevance of EZH2 inhibition in MM (Table 1). The majority of EZH2 inhibitors are selective small molecules that act as S-adenosyl-methionine (SAM)-competitive inhibitors, which is the methyl donor of methyltransferases including EZH2 [3].

**Table 1:** Pre-clinical studies of EZH2 inhibitors in MM

Author (date)	EZH2 inhibitor	Model	Main findings	Ref.
Hernando et al. (2015)	E7438 (EPZ-6438)	MM cell lines	Cells become more adherent and less proliferative with EZH2 inhibition	[18]
		Mouse model	Slower progression of the tumor, with no effect on body weight	
Agarwal et al. (2016)	UNC1999^a^ and GSK343	MM cell lines and patient samples	Reduced viability in a dose and time-dependent manner via induction of apoptosis	[23]
Zeng et al. (2017)	GSK126	MM cell lines	Decreased proliferation and increased apoptosis, reduction in stem-cell like MM cells with EZH2 inhibition	[19]
		Mouse model	Slower progression of the tumor	
Pawlyn et al. (2017)	EPZ005687 and UNC1999^a^	MM cell lines and patient samples	EZH2 inhibition reduces MM cell viability by inducing cell cycle arrest and apoptosis	[21]
Honma et al. (2017)	OR-S2^a^	MM cell lines	6 out of 8 cell lines are hypersensitive to dual EZH1/2 inhibition	[33]
Rizq et al. (2017)	UNC1999^a^	MM cell lines and patient samples	UNC1999 inhibited the growth of MM cell lines including resistant ones; cytotoxicity in MM patients cells, but not healthy donors; enhanced the cytotoxicity induced by bortezomib	[22]
		Mouse model	Reduced tumor growth; UNC1999 enhanced the cytotoxicity induced by bortezomib	
Alzrigat et al. (2017)	UNC1999^a^	MM cell lines and primary MM cells	Combining UNC1999 and BMI-1 inhibitor PTC-209 induces a significant reduction in cell viability compared to single agent	[34]
Dimopoulos et al. (2018)	EPZ-6438	MM cell lines resistant to IMiD	Combination of 5-Aza and EPZ-6438 could re-sensitize 7 of 8 cell lines to IMiD	[26]
Rastgoo et al. (2018)	EPZ-6438	MM cell lines and primary MM cells	EPZ-6438 reversed bortezomib resistance, combination with bortezomib revealed more pronounced effect on drug resistant cell lines	[17]
		Mouse model	Combination of bortezomib and EPZ-6438 significantly reduced the tumor size and prolonged the survival	
Combinations				
Neo et al. (2014)	GSK126	MM cell line (MM1S)	The dose of GSK126 required for growth inhibition and death was reduced by the addition of PTX	[36]
Harding et al. (2018)	GSK126, EPZ-6438, UNC1999	MM cell lines	Pre-treatment with EZH2 inhibitors sensitized cells to panobinostat regardless of sensitivity to single agent EZH2 inhibitor	[37]
Herviou et al. (2018)	EPZ-6438	MM cell lines and primary MM cells	EPZ-6438 reduced the number of viable cells in 9/17 patients, without correlation with EZH2 expression.	[20]
			EPZ-6438 sensitized cells to lenalidomide and pre-treatment with EPZ-6438 could overcome lenalidomide resistance in resistant cell lines	

^a^Note: OR-S2 and UNC1999 are dual EZH1/2 inhibitors

Hernando and colleagues showed that treatment of MM cell lines with EZH2 inhibitor E7438 caused decreased proliferation and increased adherence of tumor cells [18]. This was confirmed in a MM mice model where inhibition with E7438 resulted in significantly slower tumor progression based on tumor volume and weight, with no effect on mouse body weight [18]. Agarwal et al. showed that UNC1999 and GSK343 reduced viability via the induction of apoptosis in MM cell lines and primary patient samples [23]. Similar findings were obtained using GSK126 in cell lines, which inhibited growth and induced apoptosis through the mitochondrial pathway. GSK126 also acted in synergy with bortezomib to enhance apoptosis of MM cells. As mentioned earlier, the study also showed that GSK126 could eliminate stem cell-like MM cells by blocking the Wnt/β-catenin pathway. The anti-tumor effect of GSK126 was confirmed in vivo, with significant decrease in H3K27me3 and β-catenin levels by immunoblotting analysis [19].

Another study of EZH2 inhibitors EPZ005687 and UNC1999 in MM cell lines and patient samples confirmed that these molecules reduce MM cell viability in a time and concentration dependent manner by upregulating cell cycle control genes, thereby causing cell cycle arrest and apoptosis [21]. A study also tested OR-S2, a dual inhibitor of EZH1/2, in MM cell lines and found that 6 out of 8 cell lines were hypersensitive to this molecule. While EZH2 is a key factor for maintenance of global H3K27me3 levels, it is known that EZH1 can compensate when EZH2 is decreased, which the authors hypothesized could account for the greater activity of dual inhibitors. They also showed that there was no critical toxicity in rats with long-term EZH1/2 dual inhibition [33]. Dual EZH1/2 inhibitor UNC1999 was also tested in MM cell lines, patient samples and a mouse model in combination with bortezomib, demonstrating a synergistic effect compared to either treatment alone. This effect was not observed with selective EZH2 inhibitor GSK126. Using flow cytometry for Annexin V, they demonstrated that the synergy between UNC1999 and bortezomib was due to increased apoptosis [22]. UNC1999 was also shown to have synergistic and additive effect in certain MM cell lines and primary MM cells when combined with BMI-1 inhibitor PTC-209 [34].

Interestingly, EZH2 inhibitor GSK126 was able to reverse the suppression of *Runx2* and osteoblast differentiation of MC4 cells and human MM patient bone marrow stem cells induced by exposure to MM cells. These results suggest that treatment with EZH2 inhibitors could also be beneficial for the treatment of the lytic bone lesions of MM by reversing the osteoblast suppression [35].

#### Combinations of treatments

Neo et al. identified c-Rel as a regulator of EZH2 expression in MM cell lines MM1S, which expressed high levels of wild-type EZH2 [36]. They demonstrated that pentoxifylline (PTX) suppressed EZH2 expression by inhibiting c-Rel nuclear translocation. Combining PTX with EZH2 inhibitor GSK126 significantly reduced the dosage of GSK126 required to obtain a significant growth inhibition and cell death, suggesting that PTX could complement the treatment of cancers with high levels of EZH2 expression [36].

Harding and colleagues tested various EZH2 inhibitors (EPZ-6438, GSK126 and UNC1999) and found that a subset of MM cell lines were sensitive to single agent EZH2 inhibition, whereas others were resistant [37]. They found consistent changes in global H3K27 methylation in all cell lines and hypothesized that EZH2 inhibition might sensitize cells to other drugs regardless of the response to single agent EZH2 inhibitors. They demonstrated a synergistic effect when pre-treating cells with GSK126 or EPZ-6438 for several days before adding pan-HDAC inhibitor panobinostat. There was no significant difference between single agent sensitive versus resistant cell lines [37].

Similarly, Herviou et al. found a synergistic effect when combining EPZ-6438 and lenalidomide [20]. Pre-treatment of MM cells could also re-sensitize resistant cells to the effect of lenalidomide. The combination upregulates B cell transcription factors, interferes with MYC transcriptional activity and decreases Ikaros, IRF4 and MYC expression. There is also a synergistic activity altering the transcription of MM oncogenes and cell cycle genes [20].

These studies suggest that EZH2 might be used in combination with other treatment to obtain a synergistic effect.

#### Drug resistance

Dimopoulos and colleagues found that combination of 5-Aza and EZH2 inhibitor EPZ-6438, and less significantly combination of HDAC inhibitor panabinostat and EPZ-6438 could resensitize MM cell lines with acquired resistance to IMiD [26]. This process was independent of cereblon. Their findings suggested that acquires IMiD resistance is likely associated with a global epigenetic reprogramming affecting chromatin accessibility and DNA methylation. Interestingly, 5-Aza and EPZ-6438 could also increase the sensitivity of cell lines with intrinsic IMiD resistance, with similar results with EZH2 inhibition alone in some cell lines, implying that mechanisms of intrinsic resistance to IMiD might be distinct from acquired resistance [26]. This aligns with the results of Herviou et al., as described above [20].

Our team demonstrated that EPZ-6438 reversed bortezomib resistance and that combination of these two drugs could potentiate cytotoxicity in drug-resistant MM cell lines. This combination was also tested in a drug-resistant mouse xenogaft model and showed a significant reduction in tumor size compared to either drug alone and the negative control. There was significant prolonged survival without overt weight loss [17].

### Clinical trials in haematological malignancies

#### Multiple myeloma

There are a number of clinical trials involving EZH2 inhibitors in haematological malignancies (Table 2). A phase 1 clinical trial of EZH2 inhibitor GSK2816126 in various non-Hodgkin lymphomas, solid tumors and MM was terminated due to insufficient evidence of clinical activity at the maximal dose and schedule attained (NCT02082977). According to the study description, only non-Hodgkin lymphoma patients were to be included in the first stage of the study, while MM patients were to be involved in the second part of the study, therefore it appears that the drug had not yet been evaluated in MM patients [38]. To our knowledge, EZH2 inhibitors have not yet been tested in clinical trials involving MM patients.

**Table 2:** Clinical studies involving EZH2 inhibitors in haematological neoplasms

Conditions	EZH2 inhibitor	Other drugs	Phase	Trial information
B-NHL, solid advanced tumors	Tazemetostat (oral versus IV)	N/A	1	Recruiting (NCT03010982)
B-NHL and advanced solid tumors	Tazemetostat	Fluconazole, Omeprazole, Repaglinide	1	Active, not recruiting (NCT03028103)
Advanced B-NHL or solid tumors with liver dysfunction	Tazemetostat	N/A	1	Suspended (External information) (NCT03217253)
Relapsed/refractory DLBCL	Tazemetostat	Atezolizumab	1	Active, not recruiting (NCT02220842)
B-NHL, advanced solid tumors	Tazemetostat	Alone or combined with prednisolone	1/2	Recruiting; preliminary results for phase 1 [39] (NCT01897571)
RR FL and DLBCL with or without EZH2 mutation	Tazemetostat	N/A	2	Ongoing; interim results available [40, 41]
RR B-NHL with confirmed EZH2 mutation	Tazemetostat	N/A	2	Not yet recruiting (NCT03456726)
Paediatric patients with NHL, histiocytic disorder or solid tumor with *EZH2, SMARCB1* or *SMARCA4* mutation	Tazemetostat	N/A	2	Suspended (External information) (NCT03213665)
Patients previously enrolled in Tazemetostat trials	Tazemetostat	Depends on previous trial regimen	2	Recruiting (NCT02875548)
FL, DLBCL, resistant prostate cancer, RR small cell lung carcinoma	PF-06821497	N/A	1	Recruiting (NCT03460977)
B-cell lymphomas	CPI-1205	N/A	1	Active, not recruiting (NCT02395601)
Advanced DLBCL and other malignancies	MAK683	N/A	1/2	Recruiting (NCT02900651)

#### Other haematological malignancies

A majority of the ongoing clinical trials involve EZH2 inhibitor Tazemetostat (also known as E7438/EPZ-6438) [38]. A clinical trial evaluated this drug as a single agent in patients with B-cell non-Hodgkin lymphomas (B-NHL) and advanced solid tumors (Phase 1), and will investigate its use as a single agent in follicular lymphoma and diffuse large B-cell lymphoma or combined with prednisolone in DLBCL (phase 2) (NCT01897571). Recent results from the phase 1 involving 61 patients, of whom 21 had relapsed/refractory (RR) B-NHL showed a favourable safety profile and durable objective responses in 8 (38%) of these 21 patients, including complete response in 3 patients and partial response in the remaining 5 patients. Median time to first response was 3.5 months and the median duration of response was 12.4 months. The 3 patients with complete response remain on treatment with ongoing responses. Patients who responded had either activating mutations or wild-type EZH2. Interestingly, paired biopsies before and after 4 weeks of treatment in 4 patients with solid tumors revealed target inhibition of EZH2-mediated histone methylation in the tumor tissue in 3 patients. One of the on-treatment specimens showed a strong immune infiltrate that was not present at baseline or in a later specimen at progression. The authors hypothesized that EZH2 inhibition could first generate an antitumor immune response, which would then be overcome by induction of negative feedback mechanisms thereby leading to progression [39].

Interim results from a phase 2 clinical trial of Tazemetostat in RR DLBCL and FL with mutant or wild type EZH2 were published as abstracts in 2017 and 2018. The first report described the interim safety data for 165 DLBCL and FL patients, with interim efficacy results from 149 patients. The overall response rate was 40% in DLBCL with mutated EZH2 (*n* = 10) compared to 18% in patients with wild type EZH2 (*n* = 85); 63% in FL with EZH2 mutation (*n* = 8) compared to 28% in FL with wild type EZH2 (*n* = 46). 38% of mutant EZH2 FL and 30% of wild type EZH2 FL remained on study with stable disease at the time of the report. There was a favourable safety profile [40]. A second report described the results of an interim analysis of 76 FL patients. There was an overall response rate of 82%, with no patients presenting progressive disease, in patients with an activating EZH2 mutation (*n* = 22), with a median progression-free survival of more than 48 weeks and median duration of response of more than 32 weeks. As of January 2018, 56% (10/18) maintained their response and remain on study. In EZH2 wild type FL (*n* = 54), the overall response rate was 35%, with progressive disease in 30%. Median progression-free survival was more than 30 weeks and median duration of response more than 56 weeks. 58% (11/19) maintained their response. The drug was well tolerated. The authors mention previous reports of late onset response and that patients currently presenting stable disease might eventually obtain an objective response [41].

Other trials are ongoing (see Table 2). The paediatric MATCH screening trial intended to study Tazemetostat in patients with NHL, histiocytic disorders or solid tumors that harbour an *EZH2, SMARCB1* or *SMARCA4* mutation (NCT03155620), however this arm of the trial has been put on partial hold by the FDA (NCT03213665). Another Phase 1 trial of Tazemetostat in advanced B-NHL and solid tumors with liver dysfunction has been put on partial hold (NCT03217253). This is due to a report of secondary T-cell lymphoma in a paediatric patient involved in a Phase 1 trial of Tazemetostat. The patient had been on the drug for 15 months and had partial response [42].

### Response to EZH2 inhibition and anticipated mechanisms of acquired resistance

Initial results from clinical trials are encouraging; however identifying factors that are predictive of patient response is always crucial. In their study, Herviou and colleagues identified certain MM cell lines that were resistant to the effect of EPZ-6438. The effect was not correlated with levels of EZH2 expression. They identified a significant difference in CpG methylation status of inhibitor target genes between resistant and sensitive MM cell lines. Interestingly, combination with a sublethal dose of decitabine, a DNA methyltransferase inhibitor, could sensitize the resistant cell lines. Using gene expression profile, they built a score (“EZ score”) based on 15 genes deregulated by EPZ-6438, associated with H3K27me3 and of prognostic value in newly diagnosed MM patients. This score could stratify high risk and low risk patients from the HM and UAMS-TT2 cohorts. When applying this score to MM cell lines, they found a 20-fold median higher sensitivity to EPZ-6438 in cell lines with high EZ score compared to low score. Similarly, there was a correlation between EZ score and toxicity of EZH2 inhibition observed in MM patient samples [20].

Some authors have hypothesized that patients are likely to eventually develop resistance to EZH2 inhibitors similarly to other targeted therapies. In a study, diffuse large B-cell lymphoma cell line with a natural sensitivity to EZH2 inhibition were incubated with varying concentrations of EPZ-6438 until drug resistant outgrowth was observed, then a forward genetics platform was used to identify mutations conferring resistance. An acquired high frequency missense mutation in Y111D was identified in the resistant cells. Two lower frequency missense mutations, I109K and Y111N, were also identified. These mutations were located on the EZH2 D1 domain, suggesting it is a hotspot. In vitro functional assays confirmed the role of D1 domain in resistance to EZH2 inhibition. Acquisition of Y111D mutation abrogated the binding of EZH2 inhibitors to wild type and both A677G and Y641F mutant cells [43]. Other acquired missense point mutations in *EZH2*, Y111L and Y661D, were identified in DLBCL cells with acquired resistance to EZH2 inhibitor EI1. Y111L was found in wild type alleles, whereas Y661D was found only in association with gain-of-function in Y641N. These cells were also resistant to other EZH2 inhibitors. Mutation of the wild type allele alone induced an intermediate level of resistance [44].

Another study revealed enhanced IGF-1R, MAPK and P13K pathways in GSK126-resistant DLBCL cells compared to parental cells and could independently confer resistance to GSK126. Autophagy-related markers remained unchanged. Small molecule inhibitors of PI3K, IGF-1 and MEK could re-sensitize resistant cell lines to GSK126. They also identified mutations in the *EZH2* SET domain, C663Y and Y726F, that could inhibit the binding of GSK126 and confer resistance to GSK126 and EPZ-6438. Interestingly, cells with C663Y mutation remained sensitive to UNC1999, unlike cells with Y726F mutation, suggesting that changing for another EZH2 inhibitor might overcome resistance in some cases. The resistant cells were also sensitive to EED226, an allosteric inhibitor of the PRC2 complex protein EED [45].

This has implication for screening in eventual relapses on EZH2 inhibition therapy and the development of new drugs or combinations of drugs that could overcome resistance.

## Conclusions

EZH2 is involved in the pathogenesis of MM as a tumor suppressor and also has a role in drug resistance and maintenance of stem cell-like populations. Therefore, EZH2 inhibitors are gathering interest as a targeted therapy in a variety of solid and haematological malignancies. There is solid pre-clinical evidence for the relevance of EZH2 inhibitors in the treatment of MM, however further studies are needed to fully understand the mechanisms that underlie the effects of EZH2 in MM. Identifying factors that can predict response to treatment will be crucial. Certain authors have proposed a score based on gene expression profile. Further studies will be needed to validate the use of such scores, but they represent a promising avenue for selection of patients. In the past there have been instances where drugs that are effective in other haematological malignancies have show disappointing results in MM as monotherapy. However, this could be overcome by the use of drug combinations, as exemplified by clinical trials of PD-1/PD-L1 inhibitors in MM as part of multidrug regimen [46]. Pre-clinical studies highlighting the synergistic effect of EZH2 inhibitors with other chemotherapeutic drugs in MM cell lines provide interesting insight for designing potential clinical trials in MM patients. Anticipating mechanisms that could drive resistance to EZH2 inhibitors could help design new drugs or alternative treatment combinations that could help prevent or treat relapse in treated patients.

## Abbreviations

5-Aza: 5-azacytidine
ABC: Activated B-cell
AML: Acute myeloid leukemia
BMI-1: B-lymphoma Mo-MLV Insertion Region 1
B-NHL: B-cell non-Hodgkin lymphomas
CLL/SLL: Chronic lymphocytic leukemia/small lymphocytic lymphoma
EED: Embryonic ectoderm development
EZH2: Enhancer of zeste homolog 2
FL: Follicular lymphoma
GCB: Germinal-centre B-cell like
H3K27me3: Trimethylated histone H3 at lysine residue 27
IHC: Immunohistochemistry
IMiD: Immunomodulators
ITP: Idiopathic thrombocytopenic purpura
MiRNAs: MicroRNAs
MM: Multiple myeloma
PRC: Polycomb repressive complex
PTX: Pentoxifylline
RBPMS: RNA-binding protein with multiple splicing
RR: Relapsed/refractory
SAM: S-adenosyl-methionine
